# IT’S A FAMILY AFFAIR: COMMUNITY-BASED RESEARCH PARTNERSHIP TO CREATE A FAMILY-CENTERED DEMENTIA CAREGIVER PROGRAM

**DOI:** 10.1093/geroni/igac059.2708

**Published:** 2022-12-20

**Authors:** Rachel O’Conor, Dianne Oladejo, Sarah Filec, Janice Knox, Angel Griffin, Joyce Chapman, Tonya Roberson

**Affiliations:** Northwestern University, Chicago, Illinois, United States; Northwestern University, Chicago, Illinois, United States; Northwestern University, Chicago, Illinois, United States; Helping Communities Help Themselves, Chicago, Illinois, United States; Helping Communities Help Themselves, Chicago, Illinois, United States; Far South Chicago Coalition, Chicago, Illinois, United States; Governors State University, Chicago, Illinois, United States

## Abstract

People in the Black community are two times more likely to develop dementia than white people. This racial disparity is due to both social and structural factors which shape access to power, resources and exposure to health-damaging conditions by race. Due to the significant cognitive changes that occur, Black families assume substantial caregiving responsibilities to support family members, yet few caregiving programs offer culturally tailored support and training to the preferences and familial care norms of Black caregivers. In response, individuals from two community-based organizations (CBOs) and an academic medical center partnered to develop a culturally tailored, community-based, family-centered dementia caregiving research program for individuals living in far south Chicago. A team of stakeholders identified by the CBOs convened in the Fall 2021 to discuss the need for the proposed program, and together a key informant interview guide was designed. We have conducted 16 of 30 interviews and preliminary analyses of the interviews has revealed that caregivers are largely caring for parents or grandparents and are sharing caregiving responsibilities with other family members. Caregivers endorsed a need for the program; preferred a hybrid format and the need to include content related to 1) education on dementia, 2) emotional coping, and 3) linkage to medical and community-based resources. Anticipated barriers to participation included, time, location, and care for their family member while they participated. Our stakeholder team will continue to conduct key informant interviews, review and interpret data findings, and collectively develop a community-based family-centered caregiving program.

